# Investigating associations between social determinants, self-efficacy measurement of sleep apnea and CPAP adherence: the SEMSA study

**DOI:** 10.3389/fneur.2023.1148700

**Published:** 2023-07-17

**Authors:** Thibaut Gentina, Elodie Gentina, Bernard Douay, Jean-Arthur Micoulaud-Franchi, Jean-Louis Pépin, Sébastien Bailly

**Affiliations:** ^1^Ramsey General Healthcare La Louviere Hospital, Lille, France; ^2^IESEG School of Management, CNRS, UMR 9221 – LEM – Lille Economie Management, Univ. Lille, Lille, France; ^3^SANPSY, UMR 6033, University of Bordeaux, Bordeaux, France; ^4^Sleep Medicine Service, University Hospital, Bordeaux, France; ^5^HP2 Laboratory, INSERM U1300, Univ. Grenoble Alpes, Grenoble, France; ^6^EFCR Laboratory, Grenoble Alpes University Hospital, Grenoble, France

**Keywords:** obstructive sleep apnea, continuous positive airway pressure, adherence, health behavior, health literacy

## Abstract

**Study objectives:**

The prospective Self-Efficacy Measure for Sleep Apnea study (SEMSAS) is investigating thresholds for health literacy, self-efficacy and precariousness at obstructive sleep apnea (OSA) diagnosis to predict CPAP adherence. This paper describes the study protocol and presents baseline data from the ongoing study.

**Methods:**

Eligible individuals had confirmed OSA and were referred to a homecare provider for continuous positive airway pressure (CPAP) therapy initiation. Data on patient characteristics and comorbidities were collected, along with baseline evaluations of self-efficacy [15-item Self-Efficacy Measure for Sleep Apnea tool (SEMSA-15)], precariousness [Deprivation in Primary Care Questionnaire (DipCareQ)], and health literacy (Health Literacy Questionnaire). CPAP adherence over 12 months of follow-up will be determined using remote monitoring of CPAP device data. The primary objective is to define an optimal SEMSA-15 score threshold to predict CPAP adherence at 3- and 12-month follow-up.

**Results:**

Enrollment of 302 participants (71% male, median age 55 years, median body mass index 31.6 kg/m^2^) is complete. Low self-efficacy (SEMSA-15 score ≤ 2.78) was found in 93/302 participants (31%), and 38 (12.6%) reported precariousness (DipCareQ score > 1); precariousness did not differ significantly between individuals with a SEMSA-15 score ≤ 2.78 versus >2.78. Health literacy was generally good, but was significantly lower in individuals with versus without precariousness, and with low versus high self-efficacy.

**Conclusion:**

SEMSAS is the first study using multidimensional baseline assessment of self-efficacy, health literacy and precariousness, plus other characteristics, to determine future adherence to CPAP, including CPAP adherence trajectories. Collection of follow-up data is underway.

## 1. Introduction

Obstructive sleep apnea (OSA) is an important chronic condition that is characterized by repetitive complete (apnea) or partial (hypopnea) cessation of airflow due to collapse of the upper airway during sleep that induce symptoms or harms. The factors underlying these events are multifactorial and not fully understood, but are likely to include obesity, craniofacial features/changes, altered upper airway function, fluid shift towards the neck when in a supine position, and pharyngeal neuropathy (1).

OSA of at least mild severity has been estimated to affect nearly 1 billion adults aged 3–69 years worldwide (2). This is clinically relevant due to well-documented associations between OSA and several important neurocognitive, cardiovascular and metabolic comorbidities, including hypertension, cardiovascular disease, atrial fibrillation, diabetes, and even cancer (3–12). In addition, undiagnosed and untreated OSA have been associated with major depressive disorder, reduced quality of life, and increased healthcare utilization (13–19).

The standard treatment for moderate-to-severe OSA is continuous positive airway pressure (CPAP), which splints the upper airway open during sleep (20). When used correctly and for an adequate duration each night, CPAP is highly effective in suppressing sleep-related respiratory events, and improving symptoms and cognitive function (20, 21). However, in real-life clinical practice settings, the effectiveness of CPAP for suppressing apneas and hypopneas, and ameliorating the negative clinical consequences of OSA, is limited by poor adherence rates and high rates of therapy termination (22–24).

Numerous studies have investigated the clinical and physiological determinants of adherence to CPAP therapy. A high residual apnea-hypopnea index during treatment (>5–10 per hour) has been associated with poor adherence and high rates of therapy termination (25). In addition, device factors, such as the type of interface and its supply, have also been shown to influence longer-term adherence to PAP therapy (26). With respect to patient factors, higher income, educational level and number of household members have been associated with increased CPAP adherence in some studies, but currently available data are not consistent (27–29). Low socioeconomic status (SES) is another predictor of poor adherence to CPAP, and individuals with higher SES are more likely to start therapy (27, 30–32). Other factors that individually have been shown to increase the risk of non-adherence to CPAP therapy include low health literacy, forgoing healthcare, and precariousness (33–35). OSA health literacy has been found to be lower in individuals with lower educational attainment and socioeconomic status (36). Socioeconomic disparities were acknowledged as contributing to sleep health disparities and CPAP adherence in a recent American Thoracic Society consensus document (37).

A good understanding of individual characteristics at the time of the diagnosis could help to predict CPAP adherence after treatment initiation and allow clinicians and homecare providers to better manage patient adherence trajectories by selecting and implementing the most appropriate strategies to increase adherence. However, the majority of currently published studies have only investigated a single, or small number of, determinants of CPAP adherence and no one factor has been consistently identified has having high predictive value. In addition, no study has yet investigated the contribution of health literacy, precariousness and self-efficacy measures, as well as clinical characteristics, to CPAP therapy adherence.

The Self-Efficacy Measure for Sleep Apnea (SEMSA) tool is a psychometrically acceptable self-report questionnaire for the measurement of health beliefs and behaviors in individuals with OSA being treated with CPAP (38). It was developed based on Bandura’s social cognitive theory (39) and originally included 26 items (38, 39). A shorter 15-item version (SEMSA-15) was developed to improve usability in clinical practice (40) while retaining similar psychometric properties to the original version. The SEMSA study (SEMSAS) has been designed to identify specific thresholds for health literacy, self-efficacy and precariousness assessed at the time of OSA diagnosis to predict CPAP adherence over the short (3 months) and long (12 months) term. The objective of this paper is to describe the study protocol and present baseline data relating to self-efficacy based on the SEMSA-15, precariousness and health literacy from the ongoing SEMSAS, which will soon complete follow-up.

## 2. Materials and methods

### 2.1. Study design

SEMSAS is a multicenter (*n* = 3), prospective observational cohort trial (NCT04894175) that started in May 2021 and finished recruiting in December 2022. All participants are from the North of France (Bétune, Denain and Lille), a region that includes areas that have differing levels of precariousness. The study protocol was approved by the French Comité de protection des personnes Nord Ouest III (ref 2020-68). As an observational study without changes in patient care or management, potential participants were provided with information about the study. Those who did not object to the use of their data for the study were included, in accordance with French law and European General Data Protection Regulation (GDPR).

### 2.2. Study participants

Eligible individuals were adults with a physician diagnosis of OSA who were referred for initiation of CPAP therapy managed by a homecare provider. Individuals without OSA or those with OSA that was not being treated with CPAP were not eligible.

### 2.3. Data acquisition and assessments

**Figure 1:**
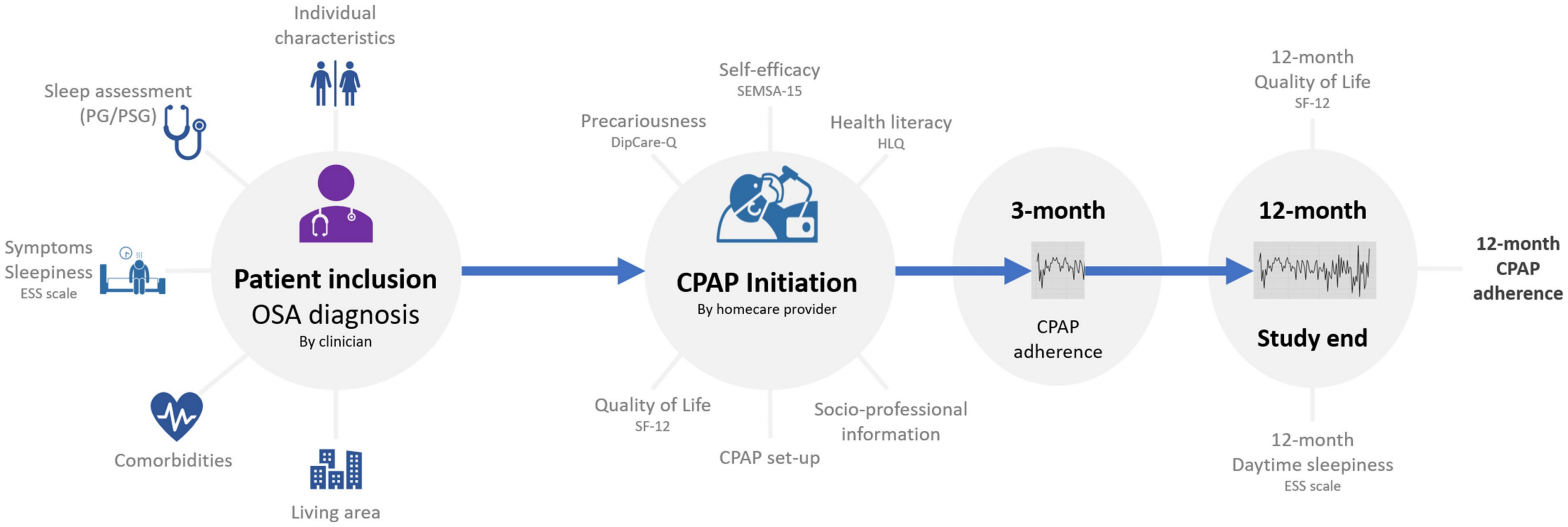
Study design and assessments. CPAP, continuous positive airway pressure; DipCare-Q, deprivation in primary care questionnaire; ESS, epworth sleepiness scale; HLQ, health literacy questionnaire; OSA; obstructive sleep apnea; PG, polygraphy; PSG, polysomnography; SEMSA-15, 15-item self-efficacy measure for sleep apnea; SF-12, Short Form-12.

Clinicians collected demographic and clinical data at baseline (after enrollment/provision of informed consent), including age, height, weight, sex, comorbidities (hypertension, cardiovascular disease, diabetes, COPD, and asthma), OSA-related symptoms (presence or absence of any of the following: severe snoring, daytime sleepiness, daytime tiredness, morning headache, and nycturia), and method used for OSA diagnosis (single night polysomnography or polygraphy). The following data were collected at CPAP initiation: device, interface, pressure, and use of a humidifier (Figure 1). Data on CPAP adherence, residual AHI and leak were collected during follow-up, and data on quality of life and the ESS score were collected at the end of follow-up (Figure 1).

Participants completed several baseline questionnaires that were provided by the homecare provider involved in setting up and initiating CPAP. These questionnaires gathered data relating to following parameters: personal characteristics (marital status, socioeconomic information, and health insurance); precariousness [the Deprivation in Primary Care Questionnaire (DipCareQ)] (41); health literacy [the Health Literacy Questionnaire (HLQ)] (42); chronotype (the degree to which individuals are active and alert at certain times of the day, primarily in the morning or evening); the shortened version of SEMSA (SEMSA-15 scale) (40); the 12-item Short Form health survey for quality of life (SF-12) (43); and the Epworth Sleepiness Scale (ESS) (44). The ESS and SF-12 will also be completed at the 12-month follow-up to allow assessment of the evolution of daytime sleepiness and quality of life during CPAP therapy.

#### 2.3.1. DipCareQ

The DipCareQ questionnaire includes 16 questions about subjective social status, education, source of income, welfare status and subjective poverty that define deprivation in three distinct dimensions: material deprivation (eight items), social deprivation (five items), and health deprivation (three items) (41). Patients provide a yes or no answer to each question, with a score of 1 for yes and 0 for no. A score for each type of deprivation is determined, then a formula is used to calculate an overall score from 0 to 5, with higher scores indicating greater deprivation. For this study, DipCareQ score groups of 0–1 and 2–5 were used.

#### 2.3.2. HLQ

The HLQ includes 44 items and covers nine distinct scales representing health literacy: (1) feeling understood and supported by healthcare providers; (2) having sufficient information to manage my health; (3) actively managing my health; (4) social support for health; (5) appraisal of health information; (6) ability to actively engage with healthcare providers; (7) navigating the healthcare system; (8) ability to find good health information; and (9) understand health information enough to know what to do (42). Responses for items in scales 1–5 are: strongly agree, disagree, agree and strongly agree. Responses for items in scales 6–9 are: cannot do, very difficult, quite difficult, easy, and very easy. Scale scores are determined by summing the item scores and then dividing by the number of items in the scale.

For scales 1–5 (four possible responses from strongly disagree to strongly agree), scores below 2.5 indicate that, on average, respondents tend to disagree with the statements within a scale. For scales 6–9 (five possible responses from cannot do/always difficult to always easy), scores below 3.5 indicate that, on average, respondents find the task within a scale difficult to do.

#### 2.3.3. SEMSA-15

The SEMSA-15 includes five items each relating to perceived risks, outcome expectancies and self-efficacy, and has been shown to have the same good psychometric properties as the 26-item version (40). Items are groups into three subscales: perception of the consequences and risks of OSA (perceived risk); perception of the expected benefits of CPAP (outcome expectations); and feeling of self-efficacy in regular use of CPAP (self-efficacy). Each item is rated on a Likert scale from 1 to 4, with higher scores indicating greater risk perception, higher benefit expectancy with treatment, and greater perceived self-efficacy (38, 39). The final score is obtained by averaging scores for each item. With the SEMSA-15, the best classification performance for prediction of CPAP adherence was for the self-efficacy sub-score, with a cut-off value of 2.78 (sensitivity 57%, specificity 79%, positive predictive value 31%, and negative predictive value of 92%) (40).

### 2.4. Validity and reliability of measures

Exploratory and confirmatory factor analyses were conducted for each measurement scale. A Structural Equation Model (AMOS 26.0) tested the measurement models among all four using full information maximum likelihood estimation (FIML) with missing values estimation. The following criteria were used to assess configural invariance: χ^2^ and degrees of freedom (χ^2^/*df* < 5), Tucker–Lewis index (TLI >0.90), comparative fit index (CFI >0.90), incremental fit index (IFI >0.90), and root mean square error of approximation (RMSEA <0.10). The reliability coefficients for all constructs were acceptable (all Cronbach alpha’s > 0.70). Convergent validity (AVE >0.50) was based on Fornell and Larcker (45).

### 2.5. CPAP adherence and follow-up

CPAP adherence will be determined from remote monitoring of device data, and will be reported at 3 and 12 months after CPAP initiation. CPAP adherence will be reported as both a continuous variable and as a binary variable (threshold of 4 h/night).

Over the 12-month follow-up period reasons for loss of follow-up will be identified to differentiate between participants who stop using CPAP and are therefore non-adherent and those who stop participating in the study but remain adherent to CPAP or those who are deceased. In addition, the number and nature of physical interventions performed by the homecare provider technician during the follow-up period will be recorded.

### 2.6. Study objectives

The primary objective of SEMSAS is to define an optimal SEMSA-15 score threshold to predict adherence to CPAP at 3- and 12-month follow-up. The study also had a number of secondary objectives (Table 1). The objective of the current report is to present full details of the study protocol and describe baseline data relating to self-efficacy based on the SEMSA-15, precariousness and health literacy for enrolled participants.

**Table 1:** Secondary study endpoints.

Secondary endpoint	Time of assessment
Define patient phenotypes based on all available baseline clinical and socio-demographic data	Baseline
Compute an overall SEMSA-15 score to predict CPAP adherence	3-month and 12-month follow-up
Determine the impact of health literacy on CPAP adherence (based on the HLQ)	3-month and 12-month follow-up
Determine the impact of precariousness on CPAP adherence (based on the DipCareQ)	3-month and 12-month follow-up
Determine the impact of patient quality of life on CPAP adherence (based on the SF-12)	3-month and 12-month follow-up
Construct an overall predictive model of CPAP adherence	3-month and 12-month follow-up
Assess interactions between CPAP adherence trajectories and patient characteristics	3-month and 12-month follow-up
Assess improvements in quality of life based on health literacy and precariousness	12-month follow-up

CPAP, continuous positive airway pressure; DipCareQ, deprivation in primary care questionnaire; HLQ, health literacy questionnaire; SEMSA-15, 15-item self-efficacy measure for sleep apnea; SF-12, short form-12.

### 2.7. Sample size

The sample size calculation was based on achieving 90% power to identify an optimal threshold for the primary objective (i.e., the SEMSA-15 score threshold to predict 3- and 12-month CPAP adherence) with a minimum area under the receiver operator characteristic curve (ROC AUC) of 0.63. Assuming a CPAP non-adherence or therapy termination rate of 25% and a 20% loss to follow-up rate of 20%, it was calculated that 300 individuals would need to be included in the study.

### 2.8. Statistical analysis

#### 2.8.1. Baseline data

In the current paper, qualitative variables are described using number and percentage, and qualitative variables as median and interquartile range (IQR). A Chi-squared test was used to compare qualitative variables and the Mann–Whitney test was used to compare quantitative variables. Effect sizes were computed using Cohen’ d coefficient for quantitative variable and phi coefficient for binary variables. Statistical analyses of baseline data were performed using SAS v9.4 (SAS Institute Inc., Cary, NC, United States). A value of *p* threshold of 0.05 was used to define statistical significance.

#### 2.8.2. Methodology for follow-up analysis

The imputation strategy for missing values will be considered based on patterns of missingness and rate of missing values. For the primary objective, predictors of CPAP adherence (as a binary variable: <4 vs. ≥4 h/night) will be determined using a multivariable mixed logistic regression model, with a random effect on center determine possible variability between centers. Various parameters, including demographic and clinical covariables, will be considered as possible confounding factors based on clinical expertise and the results of univariable analysis. ROC AUC values for different models will be compared using the Delong method to identify which has the best performance and the Youden index will be used to define the optimal threshold for the SEMSA-15 questionnaire score. The dataset will be divided into two for training and validation (75% and 25% of the total sample, respectively). The model will be developed using the training dataset and then tested on the validation dataset. Performance of the final model, including sensitivity, specificity, and positive and negative predictive values, will be computed on the validation dataset.

Trajectories of CPAP adherence will be clustered by using specific approaches for time series, such as dynamic time warping, as previously described (46). Associations between patient characteristics and CPAP adherence trajectory clusters will be investigated by using comparison tests.

Multivariable linear generalized mixed effect models will be used to study the evolution of quality of life (SF-12 score) and daytime sleepiness (ESS score) over time. Confounding factors will be selected using univariable analyses and introduced into the model. Finally, unsupervised clustering will be performed to identify specific phenotypes at the time of the diagnosis and to investigate the impact of individual determinants such as health literacy, precariousness and SEMSA-15 score on patient clinical phenotype at baseline.

Finally, a structural equation model will be considered to identify direct and indirect relationships between measured variables and 12-month CPAP adherence. This approach will allow assessment of causal relationships between different measured factors and the outcome. For this approach, exploratory and confirmatory factorial analyses will be performed for each score to assess convergent and discriminant validity. Moderating effects, including precarity and individual determinants, will be considered by performing subgroup models.

Statistical analyses will be performed using a variety of different software, including SAS, R, and AMOS.

## 3. Results

### 3.1. Study population

A total of 302 individuals were included in the study. Participant characteristics were typical of an OSA population, being predominantly male, older age and high body mass index (Table 2). There were some differences between study centers with respect to OSA diagnosis, OSA severity, rate of hypertension, and SEMSA-15 score at baseline (Supplementary Table S1); these will be corrected for in the statistical analysis of follow-up data. Hypertension was the most common comorbidity, occurring in nearly half of the study population. Based on the apnea-hypopnea index (AHI), baseline OSA was severe, and the prevalence of symptoms was high. A majority of participants reported daytime sleepiness or tiredness, almost all had severe snoring, and more than half had morning headache. Most individuals (82.5%) were married or in a permanent relationship [median duration 26 years (range 12–42)]. Nearly half of all participants (44.5%) had children living at home. More than half (58.6%) reported a professional activity (of whom 40.2% were a worker or employee), 35.8% reported being a senior manager or business owner, while 24% reported an “intermediate” profession. Validity and reliability data for all measures being used in the study are shown in Supplementary Tables S2, S3.

**Table 2:** Participant demographic and clinical characteristics at baseline, overall and in patient subgroups based on baseline 15-item self-efficacy measure for sleep apnea (SEMSA-15) score.

Characteristic	Total (*n* = 302)	SEMSA-15 score		
		Low (≤2.78) (*n* = 93; 31%)	High (>2.78) (*n* = 209; 69%)	Value of *p*^#^ (ES)*
Age, years	55 (47–64)	59 (48–68)	55 (47–63)	0.07
Male sex, *n* (%)	213 (70.5)	70 (75.3)	143 (68.4)	0.23
Body mass index, kg/m^2^	32 (28–36)	31 (28–34)	32 (28–36)	0.09
Comorbidities, *n* (%)				
Hypertension	145 (48.0)	42 (45.2)	103 (49.3)	0.51
Diabetes	42 (13.9)	18 (19.4)	24 (11.5)	0.07
Heart failure	14 (4.6)	8 (8.6)	6 (2.9)	0.03 (0.12)
Dyslipidemia	64 (21.2)	25 (26.9)	39 (18.7)	0.11
Mode of sleep apnea diagnosis, *n* (%)				
Single night polygraphy	234 (77.5)	66 (71)	168 (80.4)	0.07
Single night polysomnography	68 (22.5)	27 (29)	41 (19.6)	
Apnea-hypopnea index, /h	43 (35–57)	43 (34–55)	43 (35–58)	0.23
Oxygen desaturation index, /h	34 (23–50)	36 (27–52)	33 (23–48)	0.09
ESS score	12 (8–15)	10 (7–14)	12 (8–17)	<0.01 (0.37)
ESS score > 10, *n* (%)	170 (56)	44 (47.3)	126 (60.3)	0.04 (0.12)
OSA symptoms, *n* (%)				
Severe snoring	287 (95.0)	87 (93.5)	200 (95.7)	0.43
Daytime sleepiness	260 (86.1)	75 (80.6)	197 (94.3)	<0.01 (0.02)
Daytime tiredness	272 (90.1)	75 (80.6)	197 (94.3)	<0.01 (0.21)
Morning headache	170 (56.3)	51 (54.8)	119 (56.9)	0.73
Nycturia	206 (68.2)	62 (66.7)	144 (68.9)	0.70

Values are median (interquartile range) or number of patients (%). ^#^ Value of *p* calculated using the Mann–Whitney test for quantitative variables and Chi-square test for qualitative variables. * Effect size (ES) computed using Cohen D coefficient for continuous variables and Phi coefficient for binary variables. ESS, epworth sleepiness scale; OSA, obstructive sleep apnea; SEMSA-15, 15-item self-efficacy measure for sleep apnea.

### 3.2. Self-efficacy at baseline

The median (IQR) SEMSA-15 score at baseline was 3 (2.7–3). Overall, 31% of the study population (*n* = 93/302) had low self-efficacy based on a SEMSA-15 score of was ≤2.78 in 93 patients. Overall demographic and clinical characteristics were generally comparable between individuals with a low SEMSA-15 score compared with those who had a high SEMSA-15 score. However, those with a SEMSA-15 score of ≤2.8 were significantly more likely to have heart failure and an ESS score of >10, but significantly less likely to report daytime sleepiness or daytime tiredness, compared with individuals who had a higher SEMSA-15 score (Table 2). Although statistically significant, the effect sizes for these differences were small. Most of the fit indices for each of these measurement models were within the acceptable range suggested by Collier (47). With respect to socio-economic characteristics, individuals with a high SEMSA-15 score usually had a graduate education and were in the high socio-professional category. Of the different SEMSA-15 subscales, scores were lowest for perceived risk and highest for outcome expectations (Table 3). The total score, and scores for all three subscales were significantly higher in the high versus low SEMSA-15 score group with a large effect size (Table 3).

**Table 3:** Self-efficacy based on the SEMSA-15 score, overall and by subscale.

SEMSA-15	Total (*n* = 302)	SEMSA-15 score		
		Low (≤2.78) (*n* = 93; 31%)	High (>2.78)(*n* = 209; 69%)	Value of *p* (ES)*
Total score	3.0 (2.7–3.3)	2.5 (2.3–2.7)	3.1 (3.0–3.3)	<0.01 (2.7)
Perceived risk	2.6 (2.2–3.0)	2.0 (1.6–2.4)	2.8 (2.4–3.0)	<0.01 (1.5)
Outcome expectations	3.4 (2.8–3.6)	2.8 (2.4–3.0)	3.4 (3.2–3.8)	<0.01 (1.5)
Self-efficacy	3.2 (2.8–3.6)	2.6 (2.2–3.0)	3.4 (3.0–3.8)	<0.01 (1.7)

Values are median (interquartile range). *Effect size (ES) computed using Cohen D coefficient. SEMSA-15, 15-item self-efficacy measure for sleep apnea.

### 3.3. Precariousness at baseline

A total of 38 participants (12.6%) reported precariousness (based on a DipCareQ score > 1). Precariousness did not differ significantly between those with a SEMSA-15 score ≤ 2.78 versus >2.78, indicating that there was no association between self-efficacy and precariousness.

### 3.4. Health literacy and impact of precariousness and self-efficacy

Based on a threshold score of 2.5 for each item on the HLQ, the individuals with OSA enrolled in this study had a good level of health literacy (Table 4). Health literacy across most domains did differ significantly based on precariousness and self-efficacy, with median scores being significantly lower in patients with versus without precariousness (DipCareQ score > 1 vs. ≤1) and low versus high self-efficacy (SEMSA-15 score ≤ 2.78 vs. >2.78) (Table 4). Distributions around median values also varied between participant subgroups (Supplementary Figure S1).

**Table 4:** Health literacy item scores for the overall population and in patient subgroups based on DipCareQ index score and SEMSA-15 score.

HLQ item	All patients (*n* = 302)	Baseline DipCareQ score			Baseline SEMSA-15 score		
		0–1 (*n* = 264)	2–5 (*n* = 38)	Value of *p* (ES)*	≤2.78 (*n* = 93)	>2.78 (*n* = 209)	Value of *p* (ES)*
Feeling understood and supported by healthcare providers	3.3 (3.0–3.8)	3.3 (3.0–3.8)	3.0 (3.0–3.5)	0.02 (0.42)	3.0 (3.0–3.5)	3.3 (3.0–3.8)	<0.01 (0.48)
Having sufficient information to manage my health	3.0 (3.0–3.3)	3.0 (3.0–3.3)	3.0 (2.5–3.0)	<0.01 (0.59)	3.0 (2.8–3.0)	3.0 (3.0–3.3)	<0.01 (0.27)
Actively managing my health	2.9 (2.6–3.0)	3.0 (2.6–3.0)	2.8 (2.2–3.0)	0.01 (0.45)	3.0 (2.6–3.0)	2.8 (2.6–3.2)	0.40
Social support for health	3.2 (3.0–3.6)	3.2 (3.0–3.6)	3.0 (2.4–3.4)	<0.01 (0.71)	3.0 (3.0–3.4)	3.2 (3.0–3.6)	0.04 (0.17)
Appraisal of health information	3.8 (3.5–4.0)	3.8 (3.5–4.0)	3.5 (3.3–4.0)	0.05 (0.45)	3.0 (2.6–3.0)	3.0 (2.6–3.2)	0.03 (0.23)
Ability to actively engage with healthcare providers	4.0 (3.6–4.2)	4.0 (3.6–4.2)	4.0 (3.4–4.2)	0.07	4.0 (3.6–4.0)	4.0 (3.6–4.2)	0.03 (0.15)
Navigating the healthcare system	3.8 (3.5–4.0)	3.8 (3.5–4.0)	3.5 (3.3–4.0)	0.05 (0.33)	3.8 (3.5–4.0)	3.8 (3.5–4.2)	0.13
Ability to find good health information	3.8 (3.4–4.0)	3.8 (3.6–4.0)	3.8 (2.8–4.0)	<0.01 (0.69)	3.8 (3.4–4.0)	3.8 (3.4–4.0)	0.06
Understand health information well enough to know what to do	4.0 (3.6–4.2)	4.0 (3.6–4.2)	3.8 (3.6–4.0)	0.07	3.8 (3.6–4.0)	4.0 (3.6–4.2)	0.02 (0.25)

Values are median (interquartile range). *Effect size (ES) computed using Cohen D coefficient. DipCareQ, deprivation in primary care questionnaire; HLQ, health literacy questionnaire; SEMSA-15, 15-item self-efficacy measure for sleep apnea.

## 4. Discussion

The SEMSA study (SEMSAS) is the first to propose a multidimensional evaluation of determinants of CPAP adherence based on a combination of data including self-efficacy, precariousness, health literacy and individual characteristics/demographics. Data on these parameters will be used to predict CPAP adherence at 3 months and 1 year, and also to relate individual characteristics to CPAP adherence trajectories using remote monitoring data. It will be interesting to see associations between self-efficacy based on the SEMSA-15 score and other sociological evaluations. The inclusion and assessment of a broad range of potential factors that could influence adherence to CPAP should facilitate the identification of new predictors of CPAP adherence in conjunction with SEMSA-15, as well as confirm those already known to influence use of CPAP after therapy initiation.

Participants enrolled in SEMSAS have clinical characteristics that are indicative of a cohort with severe OSA and a clear indication for CPAP therapy. The study population showed a good level of health literacy, a low rate of precariousness, and more than two-thirds had good self-efficacy (based on a SEMSA-15 score > 2.78). Interestingly, there was no difference in precariousness between individuals with low or good self-efficacy even though other factors such as education level and profession did differ between patient subgroups based on SEMSA score (≤2.78 vs. >2.78). However, health literacy was significantly impacted by precariousness and self-efficacy, and was predictably lower in those with higher levels of precariousness and/or lower self-efficacy.

The rate of precariousness reported by study participants was 12.6%. To the best of our knowledge, SEMSAS is the first study to report precariousness in individuals starting CPAP. One previous analysis that included patients with OSA syndrome found that 43.7% reported deprivation based on the Evaluation de la précarité et des inégalités de santé dans les Centres d’examens de santé (EPICES) questionnaire score (36). Deprivation may differ from precariousness, limiting the ability to directly compare these findings. However, both studies were conducted in France and highlight the fact that precariousness and/or deprivation are likely to be important factors for a relevant proportion of individuals with OSA.

As described in the Methods section, the original SEMSA scale included 26 questions, but a shortened version was developed and validated (38) to improve clinical utility. It was this shorter version (SEMSA-15) that was used in the current study. Data on association between SEMSA-15 scores and adherence are only available from one previous study (as a secondary endpoint), where the self-efficacy subscale score was significantly correlated with mean CPAP usage at 1 month and a trend was found at 6 months (48). In addition, a number of previous prospective studies have reported an association between SEMSA-26 scores and CPAP adherence, although none had a follow-up period of longer than 3 months (28, 49–55). In addition, the SEMSA-26 score has been found to be higher in individuals defined as CPAP adherers (3.5 ± 0.52) or CPAP attempters (3.1 ± 0.7) compared with CPAP non-adherers (2.8 ± 0.2) (48).

Poor adherence to CPAP therapy remains a challenge that limits the clinical benefits of treatment in real-world settings. Identifying which variables and data are able to predict CPAP adherence is crucial both for clinicians and homecare providers. This is likely to best be achieved by collecting information at the time of CPAP initiation to allow identification of individuals that might need additional support to achieve appropriate levels of CPAP adherence during long-term therapy. Appropriate and personalized measures can then be implemented during the early stages of treatment, allowing both adherence and the clinical benefits of CPAP therapy to be maximized.

Recruitment and collection of baseline data in SEMSAS are now complete. Adherence data at 3- and 12-month follow-up are being collated, which will allow this to be correlated with the extensive baseline data collected to provide a comprehensive picture of factors associated with CPAP adherence. In addition, data on CPAP adherence are being collected daily by remote monitoring utilizing the cloud connectivity features built in to CPAP devices. This will allow additional and informative analysis of CPAP adherence trajectories rather than just at two specific timepoints during follow-up.

In conclusion, SEMSAS aims to answer specific questions to help improve knowledge about patient determinants of CPAP adherence, especially self-efficacy, precariousness and health literacy. A multidimensional evaluation of data from these assessments combined with clinical/demographic data will allow more in-depth understanding of sociological concerns that are associated with poor CPAP adherence and could limit access to healthcare. The study findings should help to facilitate the identification of individuals who will be nonadherent to CPAP therapy, and determine specific clinical thresholds for several questionnaires that might help to differentiate between those who will be adherent or non-adherent.

## Data availability statement

The raw data supporting the conclusions of this article will be made available by the authors under request.

## Ethics statement

The studies involving human participants were reviewed and approved by French Comité de protection des personnes Nord Ouest III (ref 2020-68). The patients/participants provided their written informed consent to participate in this study.

## Author contributions

TG: study conception, principal investigator, patient enrollment, interpretation of data, drafting of the manuscript, and data analysis and interpretation. EG: study conception and methodology, data management and data acquisition, and drafting of the manuscript. BD: study methodology, data acquisition and quality control, patient enrollment, and final check and approval of the manuscript. J-LP: study conception and methodology, and final check and approval of the manuscript. SB: study conception and methodology, data management, statistical analyses, and drafting of the manuscript. J-AM-F: final check and approval of the manuscript. All authors contributed to the article and approved the submitted version.

## Funding

This study was funded by AUXILAIR, France. J-LP and SB are supported by the French National Research Agency in the framework of the “Investissements d’avenir” program (ANR-15-IDEX-02) and the “e-health and integrated care and trajectories medicine and MIAI artificial intelligence” Chairs of excellence from the Grenoble Alpes University Foundation. This work has been partially supported by MIAI @ university Grenoble Alpes (ANR-19-P3IA-0003).

## Conflict of interest

The authors declare that the research was conducted in the absence of any commercial or financial relationships that could be construed as a potential conflict of interest.

## Publisher’s note

All claims expressed in this article are solely those of the authors and do not necessarily represent those of their affiliated organizations, or those of the publisher, the editors and the reviewers. Any product that may be evaluated in this article, or claim that may be made by its manufacturer, is not guaranteed or endorsed by the publisher.
