# Supplementary material for: Investigating associations between social determinants, self-efficacy measurement of sleep apnea and CPAP adherence: the SEMSA study
Source: Front Neurol. 2023 Jul 17;14:1148700. doi: 10.3389/fneur.2023.1148700 (PMC10390224; doi:10.3389/fneur.2023.1148700)

**ONLINE SUPPLEMENT**

**Investigating associations between social determinants, self-efficacy measurement of sleep apnea and CPAP adherence: the SEMSA study**

**Thibaut Gentina^1^, Elodie Gentina^2^, Bernard Douay^1^, Jean-Arthur Micoulaud^3,4^, Jean-Louis Pépin^5^, Sébastien Bailly^5*^**

^1^Ramsey General Healthcare La Louviere Hospital, Lille, France

^2^IESEG School of Management, Univ. Lille, CNRS, UMR 9221 - LEM - Lille Economie Management, F-59000 Lille, France

^3^SANPSY, UMR 6033, University of Bordeaux, Bordeaux, France

^4^Sleep Medicine Service, University Hospital, Bordeaux, France

^5^HP2 Laboratory, INSERM U1300, Univ. Grenoble Alpes, Grenoble, France; EFCR Laboratory, Grenoble Alpes University Hospital, Grenoble, France

**Table S1**. Participant demographic and clinical characteristics by enrollment center

| **Characteristics** | **Denain**  **n=38 (12.6%)** | **Bethune**  **n=122 (40.4%)** | **Lille**  **n=142 (47.0%)** | **p-value** |
| --- | --- | --- | --- | --- |
| Age, years | 54.0 [48.0; 62.0] | 56.0 [48.0; 67.0] | 55.0 [46.0; 63.0] | 0.31 |
| Male sex, n (%) | 28 (73.7) | 89 (73.0) | 96 (67.6) | 0.57 |
| Body mass index, kg/m^2^ | 32.5 [26.5; 36.0] | 31.7 [29.0; 36.0] | 31.0 [27.0; 35.0] | 0.18 |
| Comorbidities, n (%) |  |  |  |  |
| Hypertension | 15 (39.5) | 69 (56.6) | 61 (43.0) | 0.05 |
| Diabetes | 6 (15.8) | 22 (18.0) | 14 (9.9) | 0.15 |
| Heart failure | 0 (0) | 11 (9.0) | 3 (2.1) | 0.01 |
| Dyslipidemia | 12 (31.6) | 28 (23.0) | 24 (16.9) | 0.12 |
| Single night polygraphy, n (%) | 26 (68.4) | 38 (31.1)^*^ | 4 (2.8)^*#^ | <0.01 |
| Apnea-hypopnea index, /h | 38.8 [32.0; 48.0] | 45.9 [35.3; 65.4]^*^ | 42.4 [35.0; 53.0] | 0.01 |
| Oxygen desaturation index, /h | 21.9 [15.0; 35.2] | 43.2 [29.0; 63.8]^*^ | 31.8 [21.0; 42.0]^*#^ | <0.01 |
| Epworth Sleepiness Scale score | 12.5 [8.0; 19.0] | 12.0 [8.0; 16.0] | 10.5 [8.0; 15.0] | 0.32 |
| DipCareQ score 2–5, n (%) | 6 (15.8) | 15 (12.3) | 17 (12) | 0.81 |
| HLQ sub-scores |  |  |  |  |
| Feeling understood and supported by healthcare providers | 3.3 [3.0; 3.5] | 3.3 [3.0; 3.8] | 3.3 [3.0; 3.8] | 0.78 |
| Having sufficient information to manage my health | 3.0 [3.0; 3.3] | 3.0 [3.0; 3.3] | 3.0 [2.8; 3.3] | 0.91 |
| Actively managing my health | 3.0 [2.8; 3.0] | 2.8 [2.6; 3.0] | 2.9 [2.4; 3.2] | 0.48 |
| Social support for health | 3.3 [3.0; 3.8] | 3.2 [3.0; 3.6] | 3.1 [2.8; 3.6] | 0.25 |
| HLQ appraisal of health information | 3.0 [2.6; 3.2] | 3.0 [2.4; 3.2] | 3.0 [2.6; 3.2] | 0.96 |
| Ability to actively engage with healthcare providers | 4.0 [3.8; 4.2] | 4.0 [3.6; 4] | 4.0 [3.6; 4.4] | 0.18 |
| Navigating the healthcare system | 3.9 [3.5; 4.0] | 3.8 [3.5; 4.0] | 3.8 [3.3; 4.2] | 0.64 |
| Ability to find good health information | 4.0 [3.4; 4.0] | 3.8 [3.4; 4.0] | 3.8 [3.4; 4.0] | 0.69 |
| Understand health information well enough to know what to do | 4.0 [3.6; 4.0] | 3.8 [3.6; 4.0] | 4.0 [3.6; 4.2] | 0.37 |
| SEMSA-15 scores |  |  |  |  |
| Perceived risk | 2.4 [2.0; 2.8] | 2.6 [2.0; 2.8] | 2.6 [2.2; 3.0]^*#^ | <0.01 |
| Outcome expectations | 3.0 [2.4; 3.6] | 3.2 [2.8; 3.6] | 3.4 [3.0; 3.6] | 0.03 |
| Self efficacy | 3.0 [2.4; 3.6] | 3.0 [2.6; 3.6] | 3.2 [2.8; 3.8]^#^ | 0.01 |
| Total score | 2.8 [2.5; 3.3] | 2.9 [2.7; 3.2] | 3.1 [2.8; 3.3]^*#^ | <0.01 |
| High SEMSA-15 score, n (%) | 22 (57.9) | 76 (62.3) | 111 (78.2)^*#^ | <0.01 |

Values are median [interquartile range] or number of patients (%).

DipCareQ, Deprivation in Primary Care Questionnaire; HLQ, Health Literacy Questionnaire; SEMSA-15, 15-item Self-Efficacy Measure for Sleep Apnea.

P-value: overall p-value for comparison between centers.

*Significant difference between Bethune and Denain after correction for multiple tests

^#^Significant difference between Lille and Bethune after correction for multiple tests.

**Table S2.** Results of measurement models

|  | **χ^2^** | ***df*** | ***p-value*** | **χ^2^/*df*** | **CFI** | **IFI** | **TLI** | **RMSEA** |
| --- | --- | --- | --- | --- | --- | --- | --- | --- |
| **Measurement model on the whole sample**  Reflective DipCareO 16-item, 3-factor  Reflective HLQ 44-item, 9-factor  Reflective SEMSA 15-item, 3-factor  Reflective SF-12 12-item, 1-factor  Reflective ESS 8-item, 1-factor  Reflective Chronotype 7-item, 1-factor | 1,868.72  2,059.29  414.06  157.96  80.08  14.26 | 658  783  120  44  20  5 | <0.01  <0.01  <0.01  <0.01  <0.01  <0.01 | 2.84  2.63  3.45  3.59  4.00  2.85 | 0.90  0.90  0.90  0.90  0.92  0.96 | 0.90  0.91  0.90  0.91  0.92  0.96 | 0.91  0.91  0.90  0.91  0.92  0.93 | 0.07  0.06  0.07  0.07  0.06  0.06 |

Because DipCareQ is a measurement scale with dichotomous answers (yes/no), we used Robust Weighted Least Squares approach (WLSMV).

χ^2^, Chi-squared; CFI, comparative fit index; df, degree of freedom; IFI, incremental fit index; RMSEA, root mean square error of approximation; TLI, Tucker-Lewis index.

**Table S3.** Reliability and Validity of Variables

|  | **Cronbach’s α** | **Convergent Validity (AVE)** |
| --- | --- | --- |
| **DipCareQ**  1.Material deprivation  2.Social deprivation  3.Health deprivation | 0.73  0.87  0.70  0.71 | 0.51  0.52  0.50  0.50 |
| **HLQ**  1. Feeling understood and supported by healthcare providers  2. Having sufficient information to manage my health  3. Actively managing my health  4. Social support for health  5. Appraisal of health information  6. Ability to actively engage with healthcare providers  7. Navigating the healthcare system  8. Ability to find good health information  9. Understand health information enough to know what to do | 0.95  0.70  0.75  0.80  0.71  0.77  0.72  0.87  0.77  0.77 | 0.52  0.50  0.51  0.52  0.51  0.52  0.50  0.53  0.51  0.51 |
| **SEMSA-15 item**  1. Perception of the consequences and risks of OSA (perceived risk)  2. Perception of the expected benefits of CPAP (outcome expectations)  3. Feeling of self efficacy in regular use of CPAP (self efficacy) | 0.82  0.74  0.70  0.81 | 0.51  0.50  0.51  0.50 |
| **SF-12** | 0.78 | 0.51 |
| **ESS** | 0.84 | 0.50 |
| **Chronotype** | 0.71 | 0.51 |

AVE, average variance extracted; DipCareQ, Deprivation in Primary Care Questionnaire; ESS, Epworth Sleepiness Scale; HLQ, Health Literacy Questionnaire; SEMSA-15, 15-item Self-Efficacy Measure for Sleep Apnea; SF-12, Short Form-12.

**Figure S1.** Distribution of the nine Health Literacy Questionnaire (HLQ) sub-scores in patient subgroups based on baseline 15-item Self-Efficacy Measure for Sleep Apnea (SEMSA-15) threshold (dashed line indicates the median value for each sub-score).


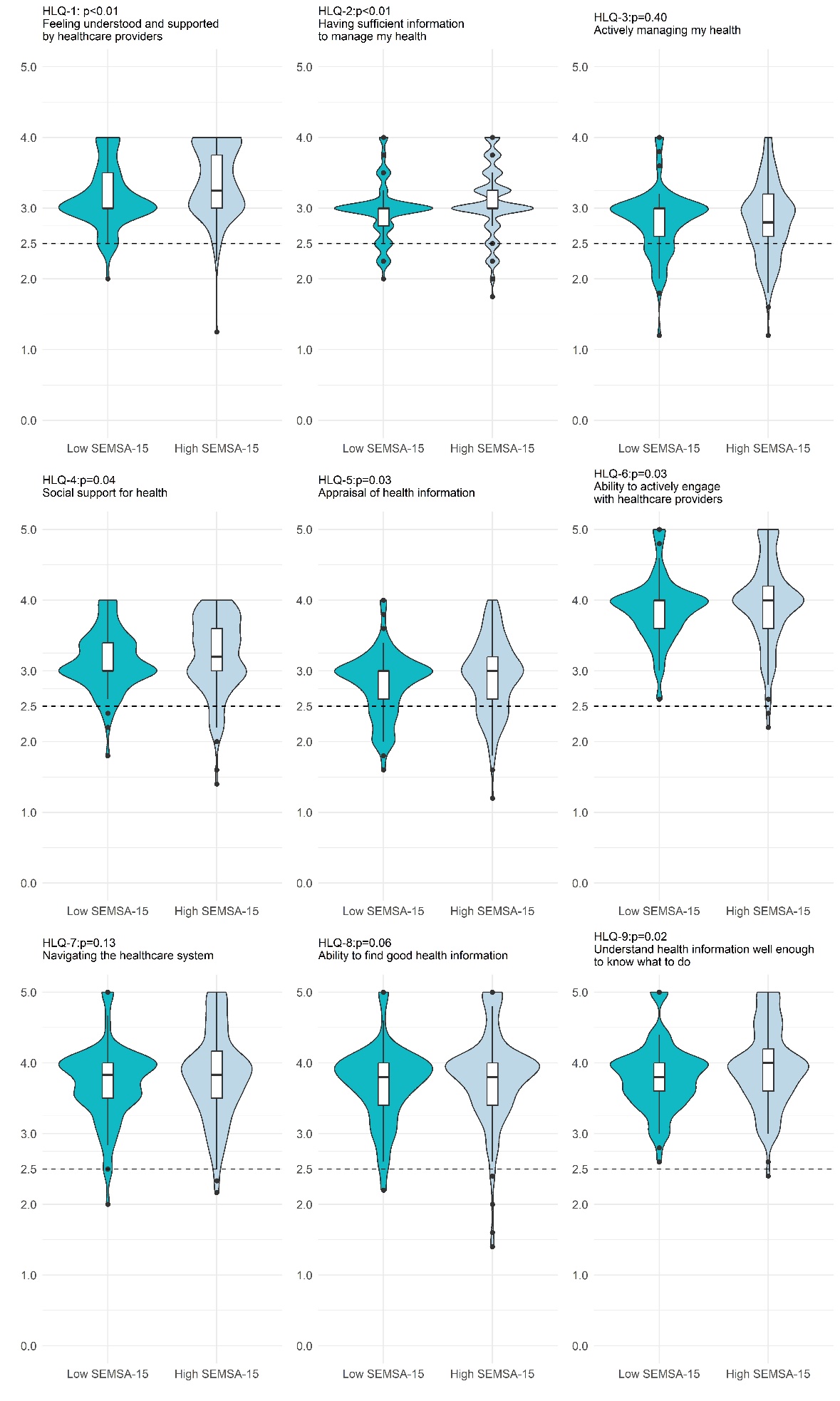

Supplement: Supplementary file 1 [file Table_1.DOCX]
